# Supplementary material for: Finite element-based nonlinear dynamic optimization of nanomechanical resonators
Source: Microsyst Nanoeng. 2025 Jan 21;11:16. doi: 10.1038/s41378-024-00854-7 (PMC11746933; doi:10.1038/s41378-024-00854-7)
Supplement: Supplementary file 1 — Supplemental Material [file 41378_2024_854_MOESM1_ESM.pdf]

Supplementary Information: Finite Element-based Nonlinear Dynamic Optimization of Nanomechanical Resonators

Zichao Li*[1], Farbod Alijani[1], Ali Sarafraz[1], Minxing Xu[1, 2], Richard A. Norte[1, 2], Alejandro M. Aragón[1], and Peter G. Steeneken*[1, 2]

[1]Department of Precision and Microsystems Engineering, Delft University of Technology, Mekelweg 2, 2628 CD Delft, The Netherlands
[2]Kavli Institute of Nanoscience, Delft University of Technology, Lorentzweg 1, 2628 CJ Delft, The Netherlands

December 4, 2024

Supplementary Note 1: Particle swarm optimization

Here, we elaborate the details in our optimizer as shown in Supplementary Figure 1, which is based on the algorithms initiated by [1, 2]. For single objective function, the velocity of the ith particle of the $(j+1)$th generation is decided by three parts, respectively [1]:

$$v_i^{(j+1)} = C_0 v_i^{(j)} + C_1 \left(PBest_i^{(j)} - x_i^{(j)} \right) + C_2 \left(GBest^{(j)} - x_i^{(j)} \right), \tag{S1}$$

where $v_i^{(j)}$ and $x_i^{(j)}$ are the velocity and position of the ith particle at the jth generation, $PBest_i^{(j)}$ is the best design that the ith particle has encountered so far, $GBest^{(j)}$ is the best design among particles in the jth generation. C_0 is the inertia weight, C_1 and C_2 are acceleration coefficients. C_0, C_1 and C_2 are all set as 2. Accordingly, we can update the design of the ith particle at the $(j+1)$th generation as

$$x_i^{(j+1)} = x_i^{(j)} + v_i^{(j+1)}. \tag{S2}$$

In order to deal with multiple objectives, the concept of Pareto dominance is introduced [2]. A particle is Pareto optimal (nondominated) if there exists no other particles in the same generation that would improve some objectives without causing a simultaneous derogation in at least one other objective. According to the Pareto dominance, we selected nondominated particles from each generation and stored them in an external repository, which is updated for each iteration. The velocity of the ith particle of the $(j+1)$th generation is derived based on

$$v_i^{(j+1)} = C_0 v_i^{(j)} + C_1 \left(PBest_i^{(j)} - x_i^{(j)} \right) + C_2 \left(Rep^{(j)} - x_i^{(j)} \right), \tag{S3}$$

where $Rep^{(j)}$ is one nondominated particle chosen from the repository. The design space is divided into hypercubes and the nondominated particles fall into different hypercubes randomly. The hypercubes with more nondominated particles are assigned a lower weight that are less likely to be selected according to roulette-wheel selection. This promotes a more equidistantly distributed nondominated particles along the Pareto front.

Besides, to reduce the drawbacks of PSO that it may converge to a local minimum due to a high convergence speed, a mutation operator is introduced after the design update of all particles. The mutation rate is designed to drop as the generation increases, which guarantees a high exploration of the whole design space at the beginning of the optimization.

Supplementary Table 1: Values and corresponding geometric design parameters for Q and β in Fig. 2

Dynamical properties	Values	L_s (µm)	w_s (µm)	θ (rad)	h (nm)
Optimized Q from PSO	3.05×10^6	83.51	6.87	0.06	40.00
Maximum Q from parametric study	3.15×10^6	100.00	7.00	0.06	40.00
Optimized β from PSO	$1.18 \times 10^{24} (m^{-2}s^{-2})$	10.00	7.00	0.03	340.00
Maximum β from parametric study	$1.18 \times 10^{24} (m^{-2}s^{-2})$	10.00	7.00	0	340.00

In Supplementary Table 1, we list the values and corresponding geometric design parameters of both Q-factor and the mass-normalized Duffing constant β obtained by PSO and by searching the parametric study results in Fig. 2 of the main text.

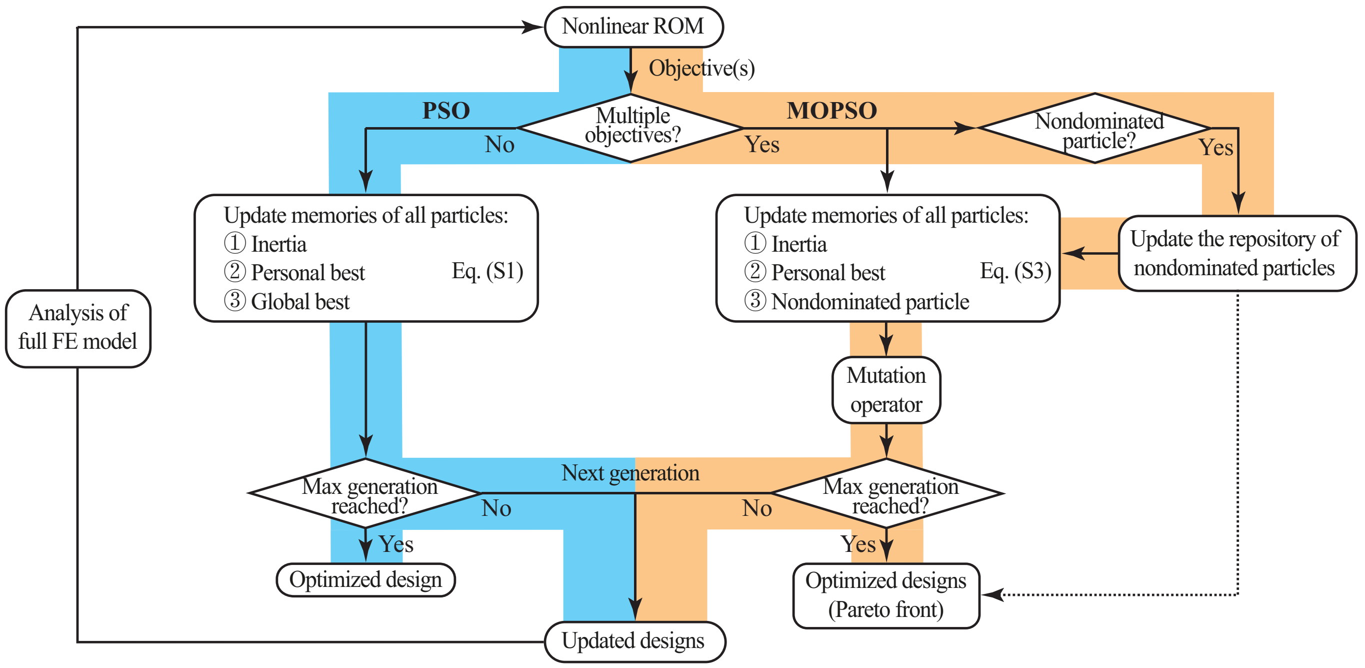

Supplementary Figure 1: **Details of the optimizer in Fig. 1.** According to the amount of objective functions, two routes based on particle swarm optimization (PSO) are available, respectively. A repository for nondominated particles is built additionally for multi-objective particle swarm optimization (MOPSO).

Supplementary Note 2: Fitting the backbone of multiple frequency response curves

Here, we elaborate on our fitting techniques for determining the fundamental resonance frequency f_0, Q-factor and the mass-normalized Duffing constant β of the string resonators from response curves at various drive levels. It is important to highlight that the resonance frequencies of nanomechanical resonators with high stress are very sensitive to changes in temperature. Thermal variations induced by both the measurement laser and the ambient environment can lead to drifts in the resonance frequencies of our resonators, sometimes by several tens of hertz, observed during consecutive frequency sweep measurements [6]. Consequently, estimating β from nonlinear response curves with varying values of f_0 becomes challenging.

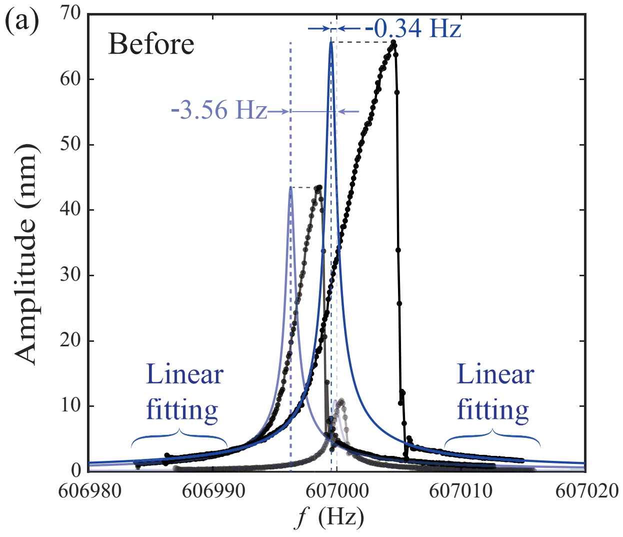

Supplementary Figure 2: **Fitting the backbone of multiple frequency response curves.** We choose three frequency sweeps (black lines with dots) from the string resonator with $w_s = 1\,\mu$m, $L_s = 90\,\mu$m and $\theta = 0.2\,$rad to demonstrate the methodology. The red curve shows the backbone fitted by multiple frequency sweeps after adjusting the resonance frequency. The blue curves show the linear fits using the off-resonance response of the nonlinear resonance curves at different drive levels.

To address this issue we fit the nonlinear frequency resonance curves as follows. For each frequency sweep, we use a Zurich Lock-in Amplifier to record the resonator amplitude at 300 driving frequency f points near the resonance frequency f_0. From each of the nonlinear frequency response curves the peak amplitude $q_{\mathrm{max,nl}}$ is determined. Then the off-resonance frequency points far below and far above the resonance frequency are fitted by Eq. (2) in the main text, with $q_{\mathrm{max,l}} = q_{\mathrm{max,nl}}$ to obtain the blue curves in Supplementary Figure 2a, along with the resonance frequency f_0 and Q factor. This procedure gives a good estimate of these parameters because the effect of the Duffing nonlinearity β far from resonance (large $|f - f_0|$) is negligible due to small q_{d}^3, and because it follows from Eq. (1) in the main text that the peak amplitude $q_{\mathrm{max,nl}}$ in the nonlinear regime is identical to the amplitude $q_{\mathrm{max,l}}$ we assumed for a linear oscillator driven at the same driving force while exhibiting no nonlinearities ($\beta = 0$).

Using the obtained values of f_0 we plot all frequency response curves at varying drive levels along the $f - f_0$ axis to align their resonance frequencies, as shown in Supplementary Figure 2b. We fit the red backbone curve Eq. (3) from the main text through the points $q_{\mathrm{max,nl}}$, $f_{max} - f_0$ in that figure to estimate β. This technique enables accurate extraction of β from multiple frequency response curves at different drive levels. Moreover, we note that the extracted values of Q are identical within 5%, indicating that it is a reasonable assumption to neglect nonlinear damping in Eq. (2) of the main text.

Supplementary Note 3: Validation of MOPSO

We generate reference Pareto fronts from parametric studies according to the Pareto criterion mentioned in Supplementary Note 1, as shown in Supplementary Figure 3. We evenly discretize the design space $L_{\mathrm{s}} = 10 \sim 100\,\mu\mathrm{m}$, $\theta = 0 \sim 0.4\mathrm{rad}$, $w_{\mathrm{s}} = 1 \sim 7\,\mu\mathrm{m}$ and $h = 40 \sim 340\,\mathrm{nm}$ and build the 1-dof nonlinear reduced-order model according to the finite element analysis of the full model. The black lines are used in Fig. 4(a) of the main text as references for the MOPSO generated Pareto fronts.

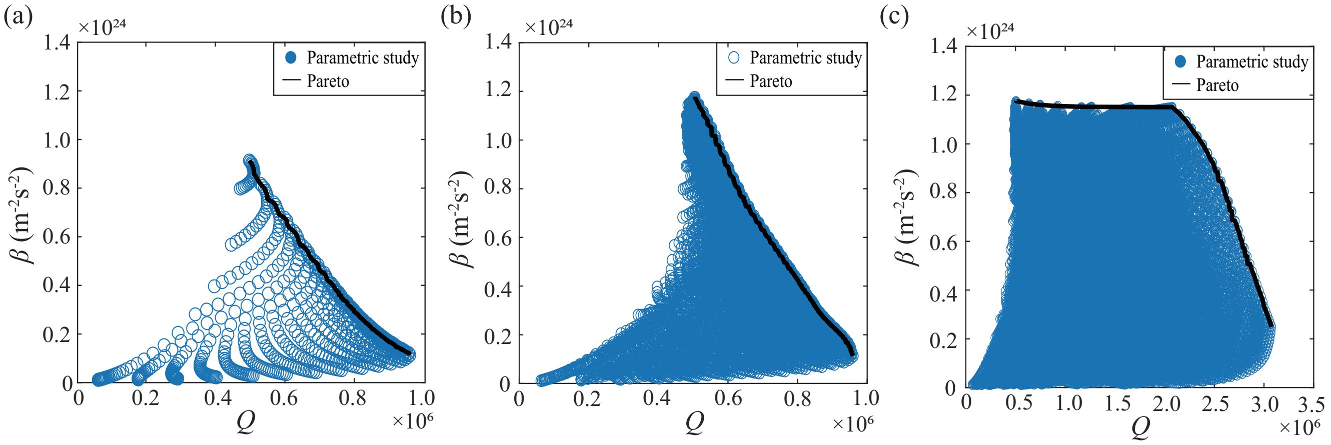

Supplementary Figure 3: **Reference Pareto fronts of different design spaces.** (a) Pareto front of varying L_{s} and θ while fixing $w_{\mathrm{s}} = 1\,\mu\mathrm{m}$ and $h = 340\,\mathrm{nm}$. (b) Pareto front of varying L_{s} ,θ and w_{s} while fixing $h = 340\,\mathrm{nm}$. (c) Pareto front of varying L_{s}, θ, w_{s} and h. The blue circles show the dynamical parameters from FE-based ROMs of devices corresponding to different design spaces.

Besides, we show the measured Q-factor and the mass-normalized Duffing constant β of our fabricated devices with constraints $w_{\mathrm{s}} = 1\,\mu\mathrm{m}$ and $h = 340\,\mathrm{nm}$. We observe that they all reside in the lower left side compared to the colored Pareto front, which proves the reliability of Pareto fronts found by MOPSO. In Supplementary Table 2, we list the geometric parameters of the measured devices, which are shown as diamonds with error bars in Fig. 4a, and their corresponding Q and β. Some of the devices listed in Supplementary Table 2 is out of the range of Fig. 4a because of low Q or β.

Supplementary Note 4: Figure-of-merits in a resonant sensor

Firstly, the response time τ_{r} demonstrates the responsivity of a resonant sensor subjected to external stimuli, which is related to the resonance frequency and the corresponding Q-factor [3]:

$$\tau_{\mathrm{r}} = \frac{Q}{\pi f_0}. \tag{S4}$$

Secondly, for a resonant sensor that operates in linear regime, the sensitivity is determined by its frequency stability that is influenced by noise. The highest signal-to-noise ratio (SNR) can be obtained by operating it at the onset of nonlinearity $a_{1\mathrm{dB}}$, where it has the largest amplitude [3, 4, 5]. Assuming the thermomechanical noise dominates the noise floor, we use Allan Deviation as the measure of frequency stability in the closed loop measurement to evaluate the sensitivity of our devices:

Supplementary Table 2: Values and corresponding geometric design parameters for Q and β in Fig. 4(a)

θ (rad)	L_s(μm)	Q	$\beta(\mathrm{m}^{-2}\mathrm{s}^{-2})$	Mark
0.4	90	671081	1.62×10^{23}	
0.4	70	561570	1.89×10^{23}	
0.4	50	588671	2.61×10^{23}	
0.4	30	558798	3.08×10^{23}	●
0.4	10	397014	8.54×10^{23}	★
0.3	90	657047	2.52×10^{23}	▲
0.2	90	804593	8.44×10^{22}	
0.2	70	698133	1.17×10^{23}	
0.2	50	668694	1.73×10^{23}	
0.2	30	597314	3.15×10^{23}	
0.2	10	483734	7.15×10^{23}	
0.1	90	587378	3.90×10^{22}	
0.1	70	541695	4.64×10^{22}	■
0.1	50	501157	7.30×10^{22}	
0.1	30	475866	1.74×10^{23}	
0.1	10	462534	6.71×10^{23}	
0	90	69112	1.38×10^{22}	
0	70	93174	2.02×10^{22}	
0	50	118166	3.68×10^{22}	
0	30	202588	1.01×10^{23}	
0	10	493902	8.15×10^{23}	

$$\sigma_y(\tau) = \frac{1}{2\sqrt{2}\ Q\ \mathrm{SNR}\sqrt{\Delta f}}\frac{1}{\sqrt{\tau}}, \tag{S5}$$

where SNR can be expressed as the ratio between the onset of nonlinearity $a_{1\mathrm{dB}}$ and the thermomechanical noise a_{th} [6]:

$$\mathrm{SNR} = \frac{a_{1\mathrm{dB}}}{a_{\mathrm{th}}} = \frac{0.9244\sqrt{\dfrac{\alpha}{Q\beta}}}{\sqrt{\dfrac{4k_{\mathrm{B}}TQ\Delta f}{m_{\mathrm{eff}}\alpha^{\frac{3}{2}}}}} = 0.4622\,(k_{\mathrm{B}}T\Delta f)^{-\frac{1}{2}}\,m_{\mathrm{eff}}^{\frac{1}{2}}Q^{-1}\alpha^{\frac{5}{4}}\beta^{-\frac{1}{2}}. \tag{S6}$$

k_{B} is the Boltzmann's constant and $\alpha = 2\pi f_0$ is the mass-normalized linear stiffness. We assume the resonator operates in room temperature $T = 298\mathrm{K}$ with the measurement bandwidth $\Delta f = 1\mathrm{Hz}$. After substituting Eq. (S6) into Eq. (S5), we can have the relationship between $\sigma_y(\tau)$ and other parameters from ROM, which is irrelevant to Q:

$$\sigma_y(\tau) = 0.765\,(k_{\mathrm{B}}T)^{\frac{1}{2}}\,m_{\mathrm{eff}}^{-\frac{1}{2}}\alpha^{-\frac{5}{4}}\beta^{\frac{1}{2}}\tau^{-\frac{1}{2}}. \tag{S7}$$

Thirdly, we define the power consumption P as the energy that is needed to be fed to the devices per second, i.e., the energy dissipated per second:

$$P = \frac{\Delta W}{T_0} = f_0\Delta W, \tag{S8}$$

where ΔW is the energy dissipated per oscillation cycle, T_0 is the period of one oscillation cycle. Considering the definition of damping ratio:

$$\zeta = \frac{1}{2Q} = \frac{\Delta W}{4\pi W}, \tag{S9}$$

and the total energy stored in the resonator supposed that it is operated at the onset of nonlinearity $a_{1\mathrm{dB}}$ for the best sensitivity:

$$W = \frac{1}{2}m_{\mathrm{eff}}\alpha a_{1\mathrm{dB}}^2, \tag{S10}$$

we can derive the power consumption P as:

$$P = \frac{2\pi f_0}{Q}\cdot\frac{1}{2}m_{\mathrm{eff}}\alpha a_{1\mathrm{dB}}^2. \tag{S11}$$

It is worth noticing that when the resonator is operated at the onset of nonlinearity, the potential energy stored in the geometric nonlinearity is several magnitudes smaller than W, that can be neglected.

Supplementary References

[1] R. C. Eberhart, Y. Shi, and J. Kennedy, Swarm intelligence (Elsevier, 2001).

[2] C. A. C. Coello, G. T. Pulido, and M. S. Lechuga, Handling multiple objectives with particle swarm optimization, IEEE Transactions on evolutionary computation 8, 256 (2004).

[3] A. Demir and M. S. Hanay, Fundamental sensitivity limitations of nanomechanical resonant sensors due to thermomechanical noise, IEEE Sensors Journal 20, 1947 (2019).

[4] A. N. Cleland, M. L. Roukes, Noise processes in nanomechanical resonators. Journal of Applied Physics 92, 2758–2769 (2002).

[5] K. L. Ekinci, Y. T. Yang, M. L. Roukes, Ultimate limits to inertial mass sensing based upon nanoelectromechanical systems. Journal of Applied Physics 95, 2682–2689 (2004).

[6] Z. Li, M. Xu, R. A. Norte, A. M. Aragón, P. G. Steeneken, and F. Alijani, Communications Physics 7, 53 (2024).
